# Effects of the lactase 13910 C/T and calcium-sensor receptor A986S G/T gene polymorphisms on the incidence and recurrence of colorectal cancer in Hungarian population

**DOI:** 10.1186/1471-2407-8-317

**Published:** 2008-11-03

**Authors:** Krisztián Bácsi, Erika Hitre, János P Kósa, Henrik Horváth, Áron Lazáry, Péter L Lakatos, Bernadett Balla, Barna Budai, Péter Lakatos, Gábor Speer

**Affiliations:** 1 First Department of Medicine, Semmelweis University, Budapest 1083 Korányi Sándor u 2/a, Hungary; 2 National Institute of Oncology, Budapest 1122 Ráth György u 7-9, Hungary

## Abstract

**Background:**

Epidemiological studies suggested the chemopreventive role of higher calcium intake in colorectal carcinogenesis. We examined genetic polymorphisms that might influence calcium metabolism: lactase (LCT) gene 13910 C/T polymorphism causing lactose intolerance and calcium-sensing receptor (CaSR) gene A986S polymorphism as a responsible factor for the altered cellular calcium sensation.

**Methods:**

538 Hungarian subjects were studied: 278 patients with colorectal cancer and 260 healthy controls. Median follow-up was 17 months. After genotyping, the relationship between LCT 13910 C/T and CaSR A986S polymorphisms as well as tumor incidence/progression was investigated.

**Results:**

in patient with colorectal cancer, a significantly higher LCT CC frequency was associated with increased distant disease recurrence (OR = 4.04; 95% CI = 1.71–9.58; p = 0.006). The disease free survival calculated from distant recurrence was reduced for those with LCT CC genotype (log rank test p = 0.008). In case of CaSR A986S polymorphism, the homozygous SS genotype was more frequent in patients than in controls (OR = 4.01; 95% CI = 1.33–12.07; p = 0.014). The number of LCT C and CaSR S risk alleles were correlated with tumor incidence (p = 0.035). The CCSS genotype combination was found only in patients with CRC (p = 0.033).

**Conclusion:**

LCT 13910 C/T and CaSR A986S polymorphisms may have an impact on the progression and/or incidence of CRC.

## Background

Colorectal cancer (CRC) was shown as the second most common of the leading causes of death from cancer in developed countries [1]. Even though colorectal tumorgenesis is a complex process, epidemiological and experimental data indicate that calcium has a chemopreventive role in the development of CRC [2]. In lactose intolerance (LI), both calcium intake and absorption are decreased [3,4]. In Finnish families, a linkage disequilibrium (LD) analysis showed a 47-kb interval locus in 2q21 chromosome region contributing to the trait of LI. Sequence analysis of this region and subsequent association analyses revealed an association between the clinically and biochemically verified LI and a C/T polymorphism in the position of 13910 of the lactase gene (LCT). The cis-acting element properties of this polymorphism, influencing the LCT gene promoter activity, may – at least partly – explain the reduced enzymatic activity of the encoded protein, called lactase or lactose-phlorizin hydrolase (LPH) [5]. Diminished LPH activity will result in LI leading to lactose malabsorption that is accompanied with decreased calcium intake [3,4].

Rasinpera et al. [6] have demonstrated that the CC genotype of the LCT gene is significantly associated with increased risk of colorectal cancer in the Finnish population but not in the British or Spanish subjects. Hungarians originate from the Finn-Ugor tribal family and the incidence of LI is in the same range in both populations (20–35%). Therefore, the potential association between the LCT 13910 C/T polymorphism and CRC in the Hungarian population could be postulated.

Calcium-sensing receptor (CaSR) is one of the key factors on cell surfaces, which by sensing the extracellular calcium concentration in the target organs responds to the changes in calcium level, thus, mediates signals towards the inside of the cell. This process is an important step in calcium homeostasis. A connection has been found between the CaSR A986S (G/T) polymorphism and serum calcium concentrations within the normal range in healthy subjects [7]. In addition, CaSR has a significant role in cell proliferation [8-10]. Reduced or loss of CaSR expression was observed in undifferentiated primary colon cancer cells compared with normal colonic epithelial cells [11]. Disruption of this ligand/receptor system may contribute to an abnormal differentiation and malignant progression. On the other hand, higher extracellular calcium through the intact CaSR might protect from CRC regulating the differentiation and proliferation of colonic epithelial cells [8-10]. The A986S polymorphism of the CaSR gene has been suggested to increase the risk of other proliferative disorders such as parathyroid adenoma [12].

In the present study, we aimed to investigate the potential relationship between these polymorphisms and the incidence and/or progression of CRC in the Hungarian population.

## Methods

### Patients

In our study, we included 538 participants, 278 subjects with primary CRC (130 female and 148 male) and 260 healthy blood donors between 2005 and 2007. Information on clinical characteristics (age at the diagnosis, sex, tumor location, TNM classification, stage groupings of the American Joint Committee on Cancer (AJCC), disease recurrence (locoregional and distant), disease-free survival (DFS), overall survival (OS), serum levels of carcinoembryonic antigen (CEA), alpha1-fetoprotein (AFP), carbohydrate antigen 19-9 (CA19-9), calcium and albumin were abstracted from medical records in oncology centers. Age at the diagnosis was 61 ± 11 years. All CRCs were histologically adenocarcinoma. Follow-up data were obtained at every 6 months. The median follow-up period was 17 months (range 1–20 months) in case of 215 patient (63 patient had no clinical follow-up data). The median DFS was 10 months (range 1–20 months). DFS was calculated for 154 patient (61 patient had metastasis at the time of the diagnosis, thus, these patients were excluded from DFS analysis). Twenty-nine participants died during the observational period. Table 1 contains the clinical and pathological data of the patients. The control group consisted of 260 healthy Caucasian blood donors without history of cancer or any chronic diseases. The enrolment criteria in the control group included the lack of gastrointestinal pain, family history for colorectal cancer and detection of blood in stool.

**Table 1:** Clinical characteristics and tumor parameters of patients according to LCT 13910 C/T and CaSR A986S genotypes

	*LCT 13910 C/T genotypes*			*CaSR A986S genotypes*		
	*TT + TC* *(n = 125)*	*CC* *(n = 90)*	*p value*	*AA + AS* *(n = 200)*	*SS* *(n = 15)*	*p value*
Age at the diagnosis (year) (mean ± SD)	60 ± 11	61 ± 10	0.54	61 ± 10	62 ± 13	0.64
Male/female (case number)	66/59	54/36	0.29	112/88	8/7	0.84
Tumor location (case number)			0.71^a^			0.40^a^
Rectum	69	52		111	10	
Left colon	27	17		41	3	
Right colon	29	21		48	2	
TNM classification (case number)						
T1	0	2	0.54^b^	2	0	0.22^b^
T2	25	13		37	1	
T3	74	60		125	9	
T4	26	15		36	5	
N0	36	25	0.87^c^	54	7	0.10^c^
N1	58	43		96	5	
N2	31	22		50	3	
M0	88	66	0.49	143	11	0.88
M1	38	23		57	4	
AJCC stages (case number)			0.87^d^			0.10^d^
I	10	6		15	1	
IIA	13	10		20	3	
IIB	13	9		19	3	
IIIA	9	4		13	0	
IIIB	26	20		44	2	
IIIC	16	18		32	2	
IV	38	23		57	4	
Serum calcium (mmol/L), (mean ± SD)	2.39 ± 0.12	2.39 ± 0.11	0.85	2.39 ± 0.11	2.39 ± 0.16	0.91
Normal range (2.2–2.6)						
Female	2.41 ± 0.11	2.36 ± 0.14	0.038^e^	2.39 ± 0.11	2.43 ± 0.18	0.42
Male	2.37 ± 0.12	2.39 ± 0.11	0.23	2.39 ± 0.11	2.35 ± 0.15	0.37
Adjusted serum calcium (mmol/L), (mean ± SD)	2.30 ± 0.11	2.30 ± 0.13	0.89	2.29 ± 0.11	2.35 ± 0.17	0.13
Normal range (2.09–2.54)						
Female	2.31 ± 0.12	2.29 ± 0.11	0.31	2.29 ± 0.11	2.40 ± 0.19	0.036^e^
Male	2.29 ± 0.10	2.31 ± 0.14	0.54	2.29 ± 0.12	2.31 ± 0.14	0.89
Serum CEA (ng/mL), [median (interquartile range)]	3.33(25.16)	3.41(13.61)	0.79	3.34(16.13)	3.01(90.77)	0.89
Normal range (< 4.3)						
Serum CA19-9 (U/mL), [median (interquartile range)]	11.15(92.09)	7.21(26.53)	0.59	9.77(42.66)	6.88(214.75)	0.98
Normal range (< 39)						
Serum AFP (ng/mL), [median (interquartile range)]	3.81(2.23)	3.43(3.05)	0.58	3.57(2.33)	3.57(6.52)	0.94
Normal range (< 13.6)						

^a^P value designates the association for rectum vs. other tumor site

^b^P value designates the association for T1+T2 vs. T3+T4 stages

^c^P value designates the association for N0 vs. N1+N2 stages

^d^P value designates the association for I-II vs. III-IV stages

^e^The association did not remain significant after adjusting for multiple testing

Detailed data were available only for 215 CRC patients

The A986S polymorphism of the CaSR gene and the 13910 C/T polymorphism of the LCT gene were investigated both in the CRC and control groups. Samples were handled anonymously in accordance with previously established ethical protocols. The study was approved by ethical committee. All subjects gave written informed consent.

### Genetic analysis of LCT 13910 C/T and CaSR A986S G/T polymorphisms

Genomic DNA was isolated from EDTA blood using a commercially available kit (Magnesil KF Genomic System, Promega, Madison, WI). LCT 13910 C/T (rs4988235) polymorphism involving a thymine (T) to cytosine (C) nucleotide change is located 13.9 kb upstream of lactase gene (LCT) in intron 13 of the adjacent minichromosome maintenance 6 (MCM6) [13]. It was genotyped with the following sequence of allele specific probes 5'-ATA AGA TAA TGT AGC CCC TGG C; 5'-ATA AGA TAA TGT AGT CCC TGG C and primers (forward) 5'-CTC TGC GCT GGC AAT ACA G and (reverse) 5'-AAA TGC AAC CTA AGG AGG AGA. The PCR mixture (20 μL) contained 1 μL genomic DNA (50 ng/μL), 0.18 μL primers (100 μmol/L; Sigma-Genosys, Woodlans, Texas), 0.04 μL probes (100 μmol/L; Applied Biosystems, Foster City, CA), 10 μL of TaqMan Universal PCR Assay Mix (Applied Biosystems) and 8.56 μL ultrapure PCR water.

Genotyping for CaSR A986S G/T (rs1801725) was performed by a Taqman Pre-Designed SNP Genotyping Assay (Applied Biosystems) [14]. This genetic variance causes an amino acid change from alanine (A) to serine (S) in the protein sequence. The PCR mixture (20 μL) for CaSR A986S polymorphism contained 1 μL genomic DNA (50 ng/μL), 0.50 μL 40× Taqman Pre-Designed SNP Genotyping Assay (Applied Biosystems), 10 μL of TaqMan Universal PCR Assay Mix (Applied Biosystems) and 8.50 μL ultrapure PCR water.

Probes were labeled at the 5' end with VIC and FAM for both polymorphisms. Cycling conditions for genotypings comprised an initial cycle at 60°C for 1 min and 95°C for 10 min, followed by 55 cycles at 92°C for 15 s and 60°C for 1 min, and a final step at 60°C for 1 min. The fluorescence intensity of was measured by the 7500 RT-PCR System (Applied Biosystems).

### Measurement of biochemical parameters (CEA, AFP, CA19-9, serum calcium and albumin)

Blood samples were obtained at the time of diagnosis of CRC. Serum tumor markers, such as CEA, AFP and CA19-9 were determined by electro-chemiluminescence immunoassays (Roche Diagnostics, Indianapolis, IN). Intra- and inter-assay coefficients of variation (CV) for all previous assays were below 4.1%, 3.8% and 2.9%, for elevated, normal and low serum concentrations, respectively. Serum calcium and albumin were determined by colorimetric assays (Roche Diagnostics). CVs were below 1.9% and 1.7% for elevated and normal serum concentrations, respectively. Calcium levels were corrected for serum albumin: corrected serum calcium = serum calcium (millimoles per liter)-0.02× [albumin (grams per liter) – 40)].

### Statistical analysis

In the primary analysis, we separately investigated the association between LCT 13910 C/T or CaSR A986S polymorphisms and disease recurrence and DFS. We examined locoregional and distant recurrences separately in the analysis between disease recurrence and genotypes carried out by binary logistic regression. Odds ratios (OR) and 95% confidence intervals (95% CI) were calculated. The events were defined as distant disease recurrence for the DFS analysis in Kaplan Meier method. The survival factor levels were compared with Log rank test. In the secondary analysis, we investigated the associations between these polymorphisms and OS by Kaplan-Meier method with Log rank test. The events were defined as death for the OS analysis.

In basic statistics, we investigated the association between the polymorphisms and cancer incidence by binary logistic regression. The distributions of the variables were analyzed by Kolmogorov-Smirnov test. Means of continuous variables with normal distribution (age at the time of diagnosis, serum calcium, DFS and OS) were compared among genotypes by analysis of variance. We used Mann-Whitney unpaired t-test or Kruskal-Wallis tests to compare the not normally distributed numerical variables (serum albumin, serum CEA, serum CA19-9, serum AFP) among genotypes. The associations between characteristics variables (gender, tumor location, TNM classification, AJCC stages) and genotypes were investigated by Chi-square test.

Analyzing the effect of LCT 13910 C/T polymorphism, we evaluated CC genotypes vs. TT+TC genotypes based on previous studies [6,15]. Additive, recessive and dominant models were used to test the associations among CaSR A986S genotypes and outcome parameters. The number of risk allele (C allele for LCT 13910 C/T polymorphism and S allele for CaSR A986S polymorphism) was used in Spearman rank correlation to investigate the effect of these two polymorphisms on variables. The Hochberg procedure was used for multiple testing adjustment, and adjusted p < 0.05 was used as the significant criterion. The results were given as mean ± standard deviation (SD) for parameters with normal distribution or median (interquartile range) for variables without normal distribution. The data were analyzed using the SPSS statistical package (SPSS Inc., Chicago, IL; version 15.0 for Windows).

## Results

**Figure 1:**
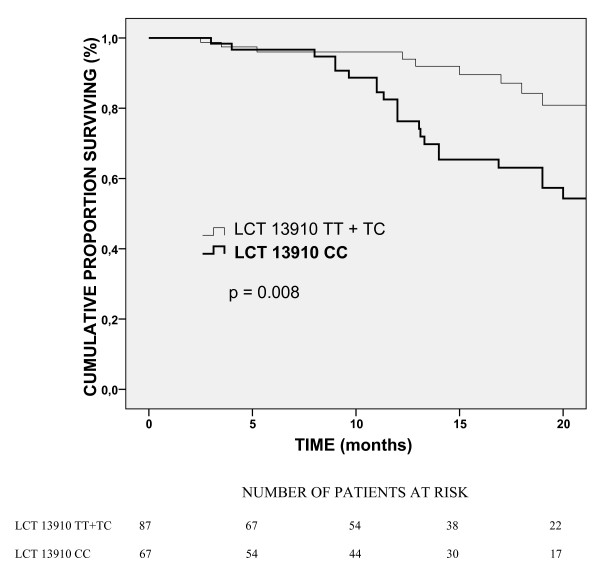
Kaplan-Meyer estimates of LCT 13910 genotypes for DFS in CRC patients. Patients with LCT 13910 CC genotype had worse outcome compared to TC + TT genotypes (log rank test p = 0.008).

Genotype frequencies followed the Hardy-Weinberg equilibrium. The distant disease recurrence (during the median 17 months follow-up period) was different in LCT genotypes: a significantly higher CC frequency was seen in patients distant disease recurrence (OR = 4.04; 95% CI = 1.71–9.58; p = 0.006) (Table 2). Also, the DFS, calculated from distant recurrence, was altered according to genotypes with reduced DFS for patient with LCT CC genotypes (log rank test p = 0.008) (Table 2 and Fig. 1). Age of patients was not different in the genotype groups (61 ± 10 years for CC genotype and 60 ± 11 years for TT + TC genotypes, p = 0.54). The locoregional disease recurrence and OS were not associated with LCT 13910 C/T genotypes (Table 2).

**Table 2:** The disease recurrences and DFS, OS according to genotypes in the patient group

	*LCT 13910 C/T*^a^			*CaSR A986S*^a^		
	*OR*	*95% CI*	*p value*	*OR*	*95% CI*	*p value*
Distant recurrence	4.04	1.71–9.58	**0.006**	1.07	0.23–4.33	0.93
Locoregional recurrence	1.37	0.49–3.87	0.55	0.63	0.08–5.19	0.66
DFS	-	-	**0.008**^b^	-	-	0.81^b^
OS	-	-	0.44^b^	-	-	0.49^b^

^a^The parameter for CC vs. TT+TC genotypes or SS vs. AA+AS genotypes were compared

^b^log rank test p value

We observed a 0.05 mmol/L difference in serum calcium level between female patients with CC genotype and with TT/TC genotypes, however it did not remain significant after correction for multiple testing. None of the other investigated laboratory and clinical variables showed any correlations with this LCT polymorphism (Table 1). Cancer incidence was not influenced by this genetic variation (OR = 1.20; 95% CI = 0.85–1.69; p = 0.30).

CaSR A986S polymorphism did not show any associations with tumor recurrence (distant and locoregional), DFS or OS in recessive (Table 2) or in additive and dominant models (data were not shown). Examining this polymorphism further, cancer incidence was found to be increased in the recessive model (OR = 4.01; 95% CI = 1.33–12.07; p = 0.042): the homozygous CaSR SS genotype was more frequent in patients than controls. This relationship was not significant when examined in dominant or additive models (data not shown). Also, the frequency of the S allele was significantly higher in CRC patients than in controls (Table 3).

**Table 3:** Genotype distributions regarding study groups

	*LCT 13910 C/T*			*CaSR A986S*		
	*Patients n = 278*	*Controls n = 260*	*p value*	*Patients n = 278*	*Controls n = 260*	*p value*
Genotype distribution (n (%))						
TT or AA	46 (16)	48 (19)		186 (67)	188 (72)	
TC or AS	119 (43)	117 (45)		75 (27)	68 (26)	
CC or SS	113 (41)	95 (36)		17 (6)	4 (2)	
C or S allele frequency	0.62	0.59	0.60	0.20	0.15	**0.025**
OR (95% CI)^a^	1.20 (0.85–1.69)		0.30	4.01 (1.33–12.07)		**0.042**
OR (95% CI) in interaction analysis (C and S alleles)			1.25 (1.03–1.52)	**p = 0.026**		

^a^The parameter for CC vs. TT+TC genotypes or SS vs. AA+AS genotypes were compared

Serum calcium concentrations were elevated in female subjects with SS genotype compared to AA/AS genotypes (Table 1), however, this correlation did not remain significant after correction for multiple testing. Other examined laboratory and clinical variables were not different among CaSR genotypes (Table 1). Serum calcium levels did not correlate with the survival parameters (DFS, OS, locoregional and distant recurrences in the follow-up period) or other laboratory values.

The number of LCT C and CaSR S risk alleles were associated with increasing tumor incidence (p = 0.035). In addition, the CCSS genotype combination (n = 5) was found only in patients with CRC (p = 0.033). Similar association was not observed with survival parameters (DFS, OS, locoregional and distant recurrences in follow-up period) or laboratory values.

## Discussion

In our study, we have found a significant correlation between LCT 13910 C/T polymorphism and the progression of CRC. Rasinpera et al. [6] found that the CC genotype was more frequent in the patient group in the Finnish population. We could not detect this association, however, CC genotype frequency was higher in the Hungarian population in general (patients: 41%, controls: 36%) than that for the Finnish population (patients: 23.5%, controls: 18%). On the other hand, we could show a decreased survival rate in CRC patients with CC genotype. This aspect was not examined in the Finnish study since it had a cross-sectional design. The reduced survival of subjects with CC genotype could not be attributed to the more advanced age of these patients since there was no difference in this respect between genotype groups.

Some authors have demonstrated that the CC genotype is associated with low LPH-specific mRNA expression and LPH activity in intestinal biopsy samples [16,17]. Also, close relationship was observed between the positive result of hydrogen breathing test and CC genotype [18]. Patients with LI consume less milk products, which is the main source of calcium [3,4]. Non-digested lactose in LI can additionally impair calcium absorption further aggravating negative calcium balance [19]. The reduction in calcium absorption can also be seen in subjects with positive breathing test but without clinical symptoms (11–32% of all lactose intolerant patients) [3,4]. In our study, subjects with CC genotype had lower serum calcium, although this correlation disappeared after correcting for multiple testing.

Increased calcium intake may reduce CRC incidence suggesting that calcium could be an important chemopreventive factor in colorectal carcinogenesis [2,10,20,21]. Reduced calcium intake will result in reduced intraluminal calcium concentration, and as a consequence, increased amounts of free bile and fatty acids. Calcium binds to bile and fatty acids, forming insoluble complexes, thus, reducing their free concentrations in the intestinal tract [22-24]. Free bile and fatty acids may promote carcinogenesis [24]. In addition, calcium itself may inhibit hyperproliferation of colonic epithelial cells [2,21,25].

Digestion of lactose by LPH will produce galactose that may also add to the pathogenetic process leading to the development of CRC, producing lower amount of galactose as enzyme product. One of the most common glycolysation abnormalities in colon cancer is the increased mucosal expression of the galactose-β-1,3-N-acetilgalactosamine, known as the Thomsen-Friedenreich blood group antigen [26]. This compound is able to bind to lectins, known factors of stimulated colon epithelial proliferation. Intestinal galactose produced by LPH enzyme has a protective effect against CRC by binding lectins, thus, inhibiting mucosal proliferation [27]. In LI, i.e. in the presence of CC genotype, the amount of intestinal galactose is decreased due to the insufficient LPH concentration. This mechanism may also contribute to the negative effect of this genotype on CRC progression observed in our study.

The CaSR A986S polymorphism appeared to have an impact on CRC incidence in our study. CaSR serves as a messenger system in controlling calcium homeostasis. CaSR is a G protein-coupled receptor [28] and it is responsible for modulating PTH and calcitonin release in response to alterations in extracellular calcium levels. Also, extracellular calcium concentration inhibits 1,25-dihidroxyvitamin D_3 _synthesis in renal proximal cells mediated by CaSR [29,30]. Higher concentration of calcium inhibits the proliferation of cancer cells [8-10]. In colonic epithelial cell lines, extracellular calcium regulates the differentiation and proliferation via the CaSR [8-10]. CaSR has been reported most highly expressed in well-differentiated regions of colon cancer and nearly lacking in poorly differentiated sites [8]. CaSR A986S polymorphism causing impaired sensing of extracellular calcium might contribute to the development of colorectal cancer. In this case, elevated extracellular calcium could not act as preventive factor on the altered CaSR. This hypothesis is strengthened by our findings that CaSR SS genotype with reduced calcium-sensing capacity was more frequent in CRC patients.

The stimulation of the CaSR may decrease colorectal carcinogenesis through several mechanisms. It promoted E-cadherin expression, a transmembran glycoprotein that functions in cell-cell adhesion and epithelial integrity [8]. Calcium/CaSR also suppress β-catenin – T cell factor (TCF) activation, which is responsible for the expression of broad range of malignant effectors, such as c-myc, gastrin, cyclin D1, cyclo-oxigenase-2 (COX-2), matrilysin, urokinase-type plasminogen activator, CD44, multidrug resistence-1 gene and its product P-glycoprotein [8,31]. On the other hand, the activation of CaSR results in cell growth and differentiation control by the mitogen-activated protein kinase (MAPK) system [10]. This stimulation is elicited through filamin-A that is bound to the intracellular region of CaSR containing the A986S mutation site [32,33]. In the mutated CaSR, stimulation of MAPK is impaired, thus, cells may proliferate in an uncontrolled manner. As a proof of this putative mechanism, SS genotype was accompanied with increased risk of CRC in our study.

According to our results, both LCT and CaSR gene polymorphisms appear to affect CRC incidence/progression. The negative effects of LCT C and CaSR S alleles could be additive since the number of copies of these disadvantageous genetic variations was accompanied with increasing incidence of CRC. In addition, CCSS genotype combination was only found in patients with CRC.

The limitations of our study include the relatively short follow-up period of 17 months, as well as the lack of detailed evaluation of calcium consumption. We did not explore underlying mechanisms either that could explain the observed correlations between the LCT/CaSR polymorphisms and the examined factors.

## Conclusion

Our results suggest that the LCT 13910 CC genotype may affect tumor recurrence and disease-free survival, while the modified calcium signaling in the CaSR 986 SS genotype could lead to increased incidence of CRC. The importance of these findings requires further investigations.

## List of abbreviations

AFP: alpha1-fetoprotein; AJCC: American Joint Committee on Cancer stages; CaSR: calcium-sensing receptor; CA19-9: carbohydrate antigen 19-9; CEA: carcinoembryonic antigen; CV: coefficient of variation; CRC: colorectal cancer; DFS: disease-free survival; LCT: lactase gene; LPH: lactose-phlorizin hydrolase; LI: lactose intolerance; MAPK: mitogen-activated protein kinase; OR: odds ratio; OS: overall survival; SD: standard deviation; TNM: tumor, node, metastasis stages; 95% CI: 95% confidence intervals.

## Competing interests

The authors declare that they have no competing interests.

## Authors' contributions

KB carried out assembly of clinical data, performed statistical analyses, genotyping and was involved in the preparation of manuscript. PL and GS have made substantial contributions to conception and design, data interpretation and have been also involved in drafting the manuscript. EH, BBudai have made contributions to acquisition and interpretation of data. JPK, HH, ÁL, BBalla also participated in genotyping. PLL participated in study coordination and helped to draft the manuscript.

## Pre-publication history

The pre-publication history for this paper can be accessed here:



## References

[B1] Capurso G, Marignani M, Delle Fave G (2006). Probiotics and the incidence of colorectal cancer: when evidence is not evident. Dig Liver Dis.

[B2] Lipkin M, Newmark H (1995). Calcium and the prevention of colon cancer. J Cell Biochem.

[B3] de Vrese M, Stegelmann A, Richter B, Fenselau S, Laue C, Schrezenmeir J (2001). Probiotics – compensation for lactase insufficiency. Am J Clin Nutr.

[B4] Carroccio A, Montalto G, Cavera G, Notarbatolo A (1998). Lactose intolerance and self-reported milk intolerance: relationship with lactose maldigestion and nutrient intake. Lactase Deficiency Study Group. J Am Coll Nutr.

[B5] Enattah NS, Sahi T, Savilahti E, Terwilliger JD, Peltonen L, Jarvela I (2002). Identification of a variant associated with adult-type hypolactasia. Nat Genet.

[B6] Rasinpera H, Forsblom C, Enattah NS, Halonen P, Salo K, Victorzon M, Mecklin JP, Jarvinen H, Enholm S, Sellick G, Alazzouzi H, Houlston R, Robinson J, Groop PH, Tomlinson I, Schwartz S, Aaltonen LA, Järvelä I, FinnDiane Study Group (2005). The C/C-13910 genotype of adult-type hypolactasia is associated with an increased risk of colorectal cancer in the Finnish population. Gut.

[B7] Marz W, Seelhorst U, Wellnitz B, Tiran B, Obermayer-Pietsch B, Renner W, Boehm BO, Ritz E, Hoffmann MM (2007). Alanine to serine polymorphism at position 986 of the calcium-sensing receptor associated with coronary heart disease, myocardial infarction, all-cause, and cardiovascular mortality. J Clin Endocrinol Metab.

[B8] Chakrabarty S, Radjendirane V, Appelman H, Varani J (2003). Extracellular calcium and calcium sensing receptor function in human colon carcinomas: promotion of E-cadherin expression and suppression of beta-catenin/TCF activation. Cancer Res.

[B9] Chakrabarty S, Wang H, Canaff L, Hendy GN, Appelman H, Varani J (2005). Calcium sensing receptor in human colon carcinoma: interaction with Ca(2+) and 1,25-dihydroxyvitamin D(3). Cancer Res.

[B10] Bhagavathula N, Kelley EA, Reddy M, Nerusu KC, Leonard C, Fay K, Chakrabarty S, Varani J (2005). Upregulation of calcium-sensing receptor and mitogen-activated protein kinase signalling in the regulation of growth and differentiation in colon carcinoma. Br J Cancer.

[B11] Sheinin Y, Kallay E, Wrba F, Kriwanek S, Peterlik M, Cross HS (2000). Immunocytochemical localization of the extracellular calcium-sensing receptor in normal and malignant human large intestinal mucosa. J Histochem Cytochem.

[B12] Miedlich S, Lamesch P, Mueller A, Paschke R (2001). Frequency of the calcium-sensing receptor variant A986S in patients with primary hyperparathyroidism. Eur J Endocrinol.

[B13] Harvey CB, Wang Y, Darmoul D, Phillips A, Mantei N, Swallow DM (1996). Characterisation of a human homologue of a yeast cell division cycle gene, MCM6, located adjacent to the 5' end of the lactase gene on chromosome 2q21. FEBS Lett.

[B14] Livak KJ (1999). Allelic discrimination using fluorogenic probes and the 5' nuclease assay. Genet Anal.

[B15] Obermayer-Pietsch BM, Bonelli CM, Walter DE, Kuhn RJ, Fahrleitner-Pammer A, Berghold A, Goessler W, Stepan V, Dobnig H, Leb G, Renner W (2004). Genetic predisposition for adult lactose intolerance and relation to diet, bone density, and bone fractures. J Bone Miner Res.

[B16] Kuokkanen M, Enattah NS, Oksanen A, Savilahti E, Orpana A, Jarvela I (2003). Transcriptional regulation of the lactase-phlorizin hydrolase gene by polymorphisms associated with adult-type hypolactasia. Gut.

[B17] Troelsen JT, Olsen J, Moller J, Sjostrom H (2003). An upstream polymorphism associated with lactase persistence has increased enhancer activity. Gastroenterology.

[B18] Buning C, Genschel J, Jurga J, Fiedler T, Voderholzer W, Fiedler EM, Worm M, Weltrich R, Lochs H, Schmidt H, Ockenga J (2005). Introducing genetic testing for adult-type hypolactasia. Digestion.

[B19] Obermayer-Pietsch BM, Gugatschka M, Reitter S, Plank W, Strele A, Walter D, Bonelli C, Goessler W, Dobnig H, Hogenauer C, Renner W, Fahrleitner-Pammer A (2007). Adult-type hypolactasia and calcium availability: decreased calcium intake or impaired calcium absorption?. Osteoporos Int.

[B20] Garland CF, Garland FC, Gorham ED (1991). Can colon cancer incidence and death rates be reduced with calcium and vitamin D?. Am J Clin Nutr.

[B21] Kallay E, Bajna E, Wrba F, Kriwanek S, Peterlik M, Cross HS (2000). Dietary calcium and growth modulation of human colon cancer cells: role of the extracellular calcium-sensing receptor. Cancer Detect Prev.

[B22] Lapre JA, De Vries HT, Termont DS, Kleibeuker JH, De Vries EG, Meer R Van der (1993). Mechanism of the protective effect of supplemental dietary calcium on cytolytic activity of fecal water. Cancer Res.

[B23] Lapre JA, Termont DS, Groen AK, Meer R Van der (1992). Lytic effects of mixed micelles of fatty acids and bile acids. Am J Physiol.

[B24] Govers MJ, Termont DS, Lapre JA, Kleibeuker JH, Vonk RJ, Meer R Van der (1996). Calcium in milk products precipitates intestinal fatty acids and secondary bile acids and thus inhibits colonic cytotoxicity in humans. Cancer Res.

[B25] Lipkin M, Newmark H (1985). Effect of added dietary calcium on colonic epithelial-cell proliferation in subjects at high risk for familial colonic cancer. N Engl J Med.

[B26] Campbell BJ, Finnie IA, Hounsell EF, Rhodes JM (1995). Direct demonstration of increased expression of Thomsen-Friedenreich (TF) antigen in colonic adenocarcinoma and ulcerative colitis mucin and its concealment in normal mucin. J Clin Invest.

[B27] Evans RC, Fear S, Ashby D, Hackett A, Williams E, Vliet M Van Der, Dunstan FD, Rhodes JM (2002). Diet and colorectal cancer: an investigation of the lectin/galactose hypothesis. Gastroenterology.

[B28] Garrett JE, Capuano IV, Hammerland LG, Hung BC, Brown EM, Hebert SC, Nemeth EF, Fuller F (1995). Molecular cloning and functional expression of human parathyroid calcium receptor cDNAs. J Biol Chem.

[B29] Brown EM (1991). Extracellular Ca2+ sensing, regulation of parathyroid cell function, and role of Ca2+ and other ions as extracellular (first) messengers. Physiol Rev.

[B30] Brown EM, Pollak M, Hebert SC (1998). The extracellular calcium-sensing receptor: its role in health and disease. Annu Rev of Med.

[B31] Wong NACS, Pignatelli M (2002). Beta-catenin – a linchpin in colorectal carcinogenesis?. Am J Pathol.

[B32] Awata H, Huang C, Handlogten ME, Miller RT (2001). Interaction of the calcium-sensing receptor and filamin, a potential scaffolding protein. J Biol Chem.

[B33] Hjalm G, MacLeod RJ, Kifor O, Chattopadhyay N, Brown EM (2001). Filamin-A binds to the carboxyl-terminal tail of the calcium-sensing receptor, an interaction that participates in CaR-mediated activation of mitogen-activated protein kinase. J Biol Chem.

